# Role of innate T cells in necrotizing enterocolitis

**DOI:** 10.3389/fimmu.2024.1357483

**Published:** 2024-02-08

**Authors:** Jianyun Liu, Sharon Joseph, Krishna Manohar, Jasmine Lee, John P. Brokaw, W. Christopher Shelley, Troy A. Markel

**Affiliations:** ^1^ Department of Surgery, Section of Pediatric Surgery, Indiana University School of Medicine, Indianapolis, IN, United States; ^2^ Riley Hospital for Children at Indiana University Health, Indianapolis, IN, United States

**Keywords:** immunity, innate T cells, NKT, MAIT, γδ T, neonates, preterm, necrotizing enterocolitis

## Abstract

Necrotizing enterocolitis (NEC) is a destructive gastrointestinal disease primarily affecting preterm babies. Despite advancements in neonatal care, NEC remains a significant cause of morbidity and mortality in neonatal intensive care units worldwide and the etiology of NEC is still unclear. Risk factors for NEC include prematurity, very low birth weight, feeding with formula, intestinal dysbiosis and bacterial infection. A review of the literature would suggest that supplementation of prebiotics and probiotics prevents NEC by altering the immune responses. Innate T cells, a highly conserved subpopulation of T cells that responds quickly to stimulation, develops differently from conventional T cells in neonates. This review aims to provide a succinct overview of innate T cells in neonates, encompassing their phenotypic characteristics, functional roles, likely involvement in the pathogenesis of NEC, and potential therapeutic implications.

## Introduction

Necrotizing enterocolitis (NEC) is a devastating disease that affects neonates born prematurely. Approximately 10% of infants are born pre-term, and about 7% of them develop NEC (1). While etiology of NEC is unknown, several risk factors have been previously reported: prematurity, very low birth weight, formula feeding, microbial dysbiosis and bacterial infection (2–7). The current treatment of NEC includes cessation of enteral feeding, use of broad-spectrum antibiotics, and parenteral administration of nutrition. About 50% of infants with NEC progress to requiring surgical intervention due to intestinal ischemia and necrosis, which is often associated with high mortality and long-term complications including intestinal stricture, short-gut syndrome, and neurodevelopmental delays (1, 8). Discovering new approaches to treat NEC medically is imperative to avoid disease progression and surgical interventions.

The diagnosis, classification, management, outcome, and complications of NEC have been summarized in many great review articles (2–6). This review focuses on exploring the possibility therapeutic role of innate T cells for NEC.

## Unique characteristics of neonatal immune response

### Tolerogenic nature

The neonatal immune system can be characterized as tolerogenic, immature, and naïve. During pregnancy, microchimerism occurs, leading to the presence of maternal cells in the fetuses and vice versa. As a result, fetal immune cells are tolerant towards maternal antigens, while maternal immune cells and antibodies are vertically transferred to offspring (9, 10). Due to this tolerogenic nature and inherent bias toward Th2-cell polarization, the developing immune system in neonates can be more susceptible to infections (11, 12).

### Immaturity and naivety

Neonatal immune cells are quantitatively fewer and qualitatively different from their adult immune cells (12, 13). As a result, maternal immune cells and antibodies circulate in offspring long after birth and impact neonatal immune responses (13–15). While maternal IgG is critical in preventing bacterial infection in neonates (16), the presence of maternal antibodies worsen the intrinsic defect of the infant’s primary antibody response but does not appear to affect their T cell response (13). Though neonatal T cells are mostly recent thymic emigrants, less antigen-experienced, and produce less IL-2 and IFN-γ (17), these cells may be more sensitive to cytokines than to antigen stimulations due to higher expression of cytokine receptors on their cell surface (18).

### Impact of microchimerism

The adaptive immune system in neonates is naïve and defective due to limited exposure to antigens and the tolerogenic environment *in utero* (19), therefore innate immunity is important in providing protection from infections (12, 20) despite its immaturity and hyporesponsiveness to stimulations (11, 21). A recent study showed that maternal microchimeric cells are enriched in fetal bone marrow and favor fetal monocyte differentiation (22), suggesting maternal/offspring microchimerism also affects neonatal innate immunity.

### Neonatal T cells have full potential

Many investigations suggest that fetuses and neonates are capable of mounting robust T cell responses (13, 19). Early in life, neonates experience rapid growth and may allocate energy towards growth rather than mounting an immune response, leading to environmental enteric dysfunction (EED). Neonates need to maintain a balance between host defense against pathogens and other essential physiological processes (17).

While significant knowledge in neonatal immunity has been acquired, little is known about neonatal innate T cells and their role in the immune responses in neonatal diseases.

## Innate T cells in neonates

### Characteristics of innate T cells

Conventional T cells express highly diverse T-cell receptors (TCRs) and respond to peptide antigens presented by polymorphic MHC class I or II molecules. In contrast, innate T cells often have limited TCR diversity and predominantly recognize non-peptide antigens presented by monomorphic non-MHC molecules (23). Many of the non-peptide antigens that activate innate T cells are microbially derived (24–31). Therefore, their development and function are changed by the microbiome (32–34).

There are three subsets of innate T cells: Natural Killer T (NKT) cells, Mucosal-Associated Invariant T (MAIT) cells and Gamma Delta (γδ) T cells. NKT and MAIT cells are mostly semi-invariant αβ T cells. All three types of innate T cells develop in the thymus and localize in non-lymphoid tissues such as the liver, lung, and intestine (24, 35).

As shown in **Table 1**, innate T cells share some common characteristics, such as their ability to bridge the innate and adaptive immune systems by quickly responding to antigens and producing large amounts of pro- and anti-inflammatory cytokines (23). The development of all three types of innate T cells is regulated by the same transcription factor, PLZF (promyelocytic leukemia zinc finger; ZBTB16) (35, 53–55). Transcriptomic analyses have demonstrated that both NKT and MAIT cells are more similar with one another compared to conventional T cells (56, 57). While all innate T cells express IL-12 receptor (IL-12R), IL-18R and other surface markers (24, 51), both NKT and MAIT cells also express NK and T cell markers (51). The similar transcriptomic profiles of NKT and MAIT cells are likely acquired by their residence in the thymus (56).

**Table 1:** Comparison of fetal/neonatal innate T cells.

	NKT cells	MAIT cells	γδ T cells	References
Maturation marker	CD45RO+ CD161+CD25+CD122+ CD127+	CD45RO+CD161+CD25+	CD27+CD28+	(36, 37)
Frequency in infants	<0.1% of CD3 T cells in cord blood	~0.1% of CD3 T cells in cord blood	~2% of CD3 T cells in cord blood	(36, 37)
Frequency in neonatal mice	Little is known	undetectable	3-4 times of αβ T cells in small intestine	(38, 39)
Microbial antigens/ligand	Microbial lipids such as α-glucuronosylceramide (GSL-1)	Microbial vitamin B metabolites	Phosphoantigens, Butyrophilins	(24, 40, 41)
Cytokine secretion	IFN-γ, IL-4	IFN-γ, IL-22	IFN-γ, TNF-α, IL-10	(42–47)
NEC impact	unknown	More MAIT cells accumulated in NEC intestines	Reduced in NEC	(48–50)
Common Characteristics	a) Limited TCR diversity; b) non-peptide antigens; c) enriched in non-lymphoid organs; d) developed in thymus; e) expressing IL-12 receptor and IL-18 receptor; f) PLZF as transcription factor; g) more cytokine production upon stimulation comparing to conventional T cells; h) proportion in T cells negatively correlates with gestational age; i) hyperproliferative potential			(23, 24, 35, 36 42–45, 51, 52)

Early in life, all three types of innate T cells seem to be more responsive and mature than conventional T cells in responding to stimulation (24, 42–44). These innate T cells are speculated to play important roles in tissue homeostasis and fighting against infection during early life when conventional T cells are still naïve and immature (58).

### Neonatal NKT cells

NKT cells are innate T cells that can be activated by lipid antigens, with CD1d, an MHC class I-like molecule, acting as the antigen presenting molecule (59–62). The most potent lipid antigen for NKT cells is alpha-Galactosylceramide (a-GalCer, KRN7000), which is a synthetic glycolipid derived from the marine sponge *Agelas mauritianus* (63, 64). NKT cells can also be activated by various endogenous and microbial lipid antigens such as iGb3, sulfatide, and α-glucuronosylceramide (GSL-1) (28, 40, 65, 66). Activated NKT cells may be utilized in vaccine development and the treatment of conditions like autoimmune diseases, graft-versus-host disease, infections, neurological diseases, and cancer (67–77).

NKT cells are either CD4^+^ or CD4^-^CD8^-^ T cells. Based on TCR usage, NKT cells can be further categorized as Type I (invariant TCR) and the much less studied Type II (variable CD1d-restricted TCR) NKT cells. In this review only type I NKT cells related work is discussed.

Studies have shown that a low number of neonatal NKT cells are present in cord blood (45, 78) and they are less responsive to stimulation compared to adult NKT cells (45). However, neonatal NKT cells were more responsive to stimulation compared to neonatal conventional T cells (45). The population of neonatal NKT cells was higher in the blood of day 14 preterm infants compared to those from age-matched full-term infants. In the subsequent 2-3 weeks, however, that higher proportion of NKT cells decreases to a level similar to that of full-term infants. This is likely due to gestational development because the proportion of CD3^+^ T cells expands and positively correlates with gestational age (52). The proportion of NKT cells in the blood should expand as infants grow older since the proportion of NKT cells in adult blood is much higher than that in cord blood (45).

Neonates have a Th-2 biased immunity with neonatal NKT cells producing more IL-4 than IFN-γ (46, 47). Interestingly, NKT cells are enriched in the fetal small intestine. These small intestinal NKT cells, different from NKT cells from other fetal organs, express mature markers and IFN-γ upon stimulation, resembling adult NKT cells (44).

As shown in **Table 1**, not much is known about the frequency of neonatal NKT cells in mice.

### Neonatal MAIT cells

MAIT cells predominantly recognize non-peptide microbial antigens presented by monomorphic MHC class I-like molecule (MR1) (23, 30, 79). MAIT cells express limited TCR diversity (Vα19 in mice, Vα7.2 in humans with limited variation of TCR-β chains). The research of MAIT cell antigens experienced a breakthrough when 5-(2-oxopropylideneamino)-6-D-ribitylaminouracil (5-OP-RU) was identified and remains the most potent MAIT cell agonist to date (31, 41, 80). MAIT cells are mostly CD8^+^ or CD4^-^CD8^-^ T cells. Emerging research demonstrates that MAIT cells are involved in many conditions, such as infection, cancer, tissue repair, autoimmunity, inflammation, and metabolic diseases (81–89).

Data on how gestational age impacts MAIT cell levels is conflicting. One study suggests that the proportion of neonatal MAIT cells is low and does not seem to be affected by gestational age ranged 23 to 28 weeks (52). However, another study using cord blood from broader range of gestational ages (24 weeks to full term) showed that the proportion of MAIT cells in CD3^+^ T cells negatively correlated with gestational age (36). Currently, there are two ways to identify human MAIT cells: CD3^+^Vα7.2^+^ CD161^high^ T and CD3^+^MR1:5-OP-RU tetramer^+^ cells. In adult blood, these two populations almost fully overlap. However, in cord blood, only a small portion of CD3^+^Vα7.2^+^ CD161^high^ T cells are also MR1:5-OP-RU tetramer^+^ (36, 53). This is likely due to specific expansion after encountering microbial antigens. Cord blood-derived MAIT cells consistently are more capable to proliferate upon stimulation compared to adult MAIT cells (36). Allogeneic hematopoietic cell transplantation study showed expansion of MAIT cells in recipients after cord blood transplantation but not in adult bone-marrow or peripheral blood stem cell transplantations, supporting the high proliferative capacity of neonatal MAIT cells (90).

Mouse MAIT cell studies have been lagging due to the scarcity of mouse MAIT cells. To solve this problem, a wild-derived inbred CAST/EiJ mouse model was discovered with frequencies of MAIT cells 20 times more than those in C57BL/6J mice (38). MAIT cells also increase significantly in the transgenic mice expressing the TCR Vα19, but its application is limited due to high non-specific binding of MR1:5-OP-RU tetramer in other T cells (91–93). Like human MAIT cells, mouse MAIT cells are almost undetectable at birth but expand significantly after encountering the developing microbiome (38).

### Neonatal γδ T cells

Most mammalian T cells express αβ TCR. A small population of T cells express gamma and delta (γδ) TCR and these cells are called γδ T cells. The antigen presenting molecule for γδ T cells is not known. γδ TCR may interact with antigens in an antibody/antigen binding fashion (24, 94, 95). The functions of γδ T cells include immune surveillance, thermogenesis, and tissue homeostasis (96). γδ T cells are known to be important for maintaining mucosal tolerance (97, 98). Although similar numbers of γδ T cells can be found in the intestine of germ-free and specific pathogen-free mice (99), the crosstalk between microbiome and γδ T cells is important for the effector function of γδ T cells (32). Removal of gut microbiome by antibiotic treatment in drinking water impairs oral tolerance and also transiently removes intestinal γδ T cells.

Neonatal γδ T cells are Th2-prone and more naïve than adult γδ T cells, but more Th1-prone compared to neonatal αβ T cells. Thus, it seems reasonable to hypothesize that neonatal γδ T cells may be key providers of immunoprotection and immunomodulation in the perinatal period (42). γδ intraepithelial lymphocytes (IEL) are the first T-cell subset present in the intestine during embryogenesis (39, 100). Neonatal mouse γδ IELs were found to produce higher levels of cytokines, such as IFN-γ and IL-10, as compared to neonatal αβ IELs and adult γδ IELs, indicating enhanced activity of γδ IELs during early life (39).

Neonatal γδ T cells are more diverse compared to adult γδ T cells. The dominant Vγ9Vδ2 subset in human adult blood is due to the post-natal expansion of cells expressing unique CDR3 formed in response to encountering phosphor-antigens derived from the microbe-specific isoprenoid synthesis pathway. During mouse embryonic development, there are waves of γδ T cell development that start as early as day 15 of gestation so most peripheral tissues are colonized by long-lived γδ T cells early in life (96). The first wave of mouse γδ T cells are dendritic epidermal T cells (DETCs). These DETCs migrate to mouse skin and proliferate there during fetal development (24, 96). While some γδ T cells can be restored in 2 weeks in adult mice, fetal γδ T cells cannot be regenerated in the adult thymus (24).

Similar to neonatal NKT cells, the proportion of neonatal γδ T cells are larger in the blood from preterm infants than that from full-term infants, and the proportion of γδ T cells decreases to a similar level as that from full-term infants in the next 2-4 weeks (52). This is likely due to the expansion of CD3^+^ T cells in late gestational stages.

## The role of innate T cells in NEC

Gut microbial community perturbations are the most consequential risk factor for NEC (101). The intestinal microbiome of preterm infants is distinct and less diverse than that of term-born infants. Interestingly, the gut microbiome in preterm infants seems to have an orderly progression where the bacterial classes switch from Bacilli to Gammaproteobacteria to Clostridia, and is minimally influenced by mode of delivery, antibiotics, or feeds (101, 102).

It is not clear how the bacterial class switch in preterm infants increases their risk of developing NEC but analysis of gene expression analysis in NEC tissues does reveal an altered immune response (48, 103–106). The microbial dysbiosis in NEC likely alters the development of innate T cells given the microbiome’s known influence on innate T cell maturation, activation, and expansion via changes in microbial antigens and modulation of the mucosal microenvironment (32, 81, 107, 108). Immune cell development needs microbial exposure, but there seems to be a “window of opportunity” (17, 58, 109). Using mouse models, several groups have demonstrated that exposure to certain microbiome early in life defines hosts’ T cell functions in adulthood (17, 58, 109). It is reasonable to speculate that the microbial dysbiosis in NEC impacts not only neonatal immunity but also long-term immunity beyond when the disease is resolved.

Studies about the relationship between innate T cells and NEC are sparse. A recent report has shown that more MAIT cells accumulate in the intestine of NEC patients compared to control infants. However, these MAIT cells within NEC intestine are mostly CD4^-^CD8^-^, while MAIT cells from healthy intestine are mainly CD8αα^+^ MAIT cells (49). CD8αα^+^ MAIT cells are known to be more mature than CD4^-^CD8^-^ or CD8αβ^+^ MAIT cells (43, 110). These results suggest that there are more immature MAIT cells residing in NEC intestines. Weitkamp et al. discovered significantly lower CD8 ^+^ γδ IEL in preterm infants with NEC compared to control infants, suggesting that γδ IELs depletion occurs during the development of NEC (39).

It is worth noting that a unique population of IELs, called innate CD8alpha (*i*CD8α) cells, that expresses the CD8αα homodimer and may be involved with NEC pathogenesis. Though neither T cells nor dendritic cells, they are IL-12R positive and responsive to stimulation by IL-12 and PMA/Ionomycin. *i*CD8α cells show capacity in antigen processing/presentation and protection from bacterial infection (111). *i*CD8α cells are also reduced in NEC patients compared to control infants, consistent with reduced CD8αα^+^ MAIT and CD8^+^ γδ T cells in NEC (39, 49, 111). These observations indicate mucosal CD8^+^ lymphocytes, either TCR^+^ (MAIT and γδ T cells) or TCR^-^ (innate lymphoid cells) may be important in preventing NEC.

**Figure 1:**
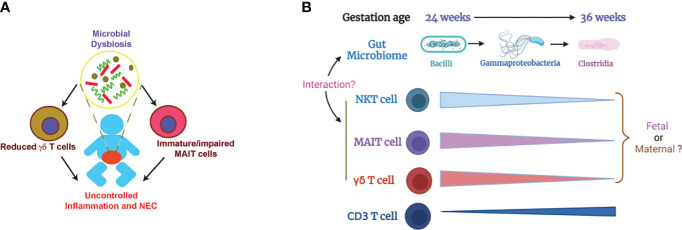
Illustration of how microbial dysbiosis and immaturity of innate T cells may affect NEC pathogenesis. **(A)** Immature/impaired MAIT cells and/or reduced γδ T cells, possibly caused by microbial dysbiosis, contribute to NEC pathogenesis (39, 48, 49, 112). **(B)** Change of gut microbiome and innate T cells in preterm infants based on gestation age. The proportions of innate T cells negatively correlate with gestational age. Little is known about whether the innate T cells in preterm infants are of fetal or maternal origin. It is also unclear how the transition of gut microbiome affects the development and function of innate T cells (36, 52, 101, 102). .

Deficiency of MR1 in neonatal mice renders protection from NEC pathogenesis (112) while TCRδ-deficient neonatal mice develop worse NEC disease compared to WT controls (39). These data suggest that innate T cells, probably altered by microbial dysbiosis, play a role in NEC pathogenesis (**Figure 1A**). Little is known how innate T cells may contribute to the pathogenesis of NEC. Innate T cells are known to bridge the innate and adaptive immune system and can mediate immune tolerance (113–116). It is speculated that the function of innate T cells may be altered with reduced immune tolerance due to microbial dysbiosis and immaturity in preterm infants. Another possible factor is IL-17 production that plays a critical role in pathogenesis of NEC (117). Innate T cells are known to produce IL-17 (118–120). Innate T cells from preterm infants may produce more IL-17 due to immaturity and microbial dysbiosis, contributing to NEC pathogenesis.

## Conclusions and future direction

The current standard treatment regimen of NEC includes cessation of enteral feeding, institution of parenteral nutrition, initiating broad-spectrum antibiotics, respiratory support, and surgical intervention as needed (1). Human breastmilk has long been utilized as a way to reduce NEC (1). The expansion of Bifidobacteriaceae in gut microbiome after birth also decreases the risk of NEC (3, 100, 121). Human milk oligosaccharide (HMO) is important to bifidobacterial colonization, consistent with the observation that breast-feeding lowers the incidence of NEC (100). Prebiotics (*e.g.* human milk oligosaccharide) and probiotics (*e.g. Bifidobacteria*) are being investigated as potential preventative and therapeutics approaches for NEC (3, 7, 121, 122). A few acting mechanisms of probiotics in preventing NEC have been proposed (123), but little is known about how prebiotics and probiotics therapies may change innate T cells in NEC.

Because of their limited TCR diversity and monomorphism, innate T cells would be an off-the-shelf cell-based therapy with minimal graft-versus-host disease (124). Innate T cells can quickly respond to antigens and produce large amounts of pro- and anti-inflammatory cytokines to bridge the innate and adaptive immune systems (23, 51, 125–127). Innate T cells can also acquire effector T cell characteristics and accumulate in mucosal tissues early in life (42–44). IL-22 has been shown to alleviate NEC (128) and innate T cells are capable of producing IL-22 (43, 118). Genetic engineering technologies, such as CRISPR/Cre and CAR-T cells (76, 129–131), may facilitate the production of IL-22-producing γδ T and/or MAIT cells, even *i*CD8α IELs, for cell-based immunotherapy for NEC.

Contrarily, the accumulation of immature innate T cells in preterm infants may lead to the development of NEC. Blocking the activation of immature innate T cells may reduce the incidence of NEC. Antibodies for CD1d and MR1, the antigen presenting molecules for NKT and MAIT cells respectively, are effective in suppressing NKT and MAIT cell activation (132–134). These MR1 and CD1d specific antibodies may also be a potential avenue for further investigations in NEC immunotherapy.

Preterm infants are not developmentally primed to be colonized by microorganisms and the normal neonatal microbial adaptation may be hazardous in preterm infants (135). It is well-established that the development and maturation of innate T cells are influenced by the microbiome. The levels of innate T cells in preterm infants negatively correlates with gestational age (**Figure 1B**, **Table 1**) but the mechanism is unknown. Because the mother and offspring form a microchimer, innate T cells in fetus and preterm infants can be from fetal or maternal origin. The gut microbiome in preterm infants is transitional and different from term infants, but it is unclear exactly how the innate T cells in preterm infants are influenced by the altered gut microbiome. Future work should focus on identifying the origin of fetal and neonatal innate T cells and how their development and function are impacted by beneficial or pathogenic microbiome. The knowledge gained from this work will help facilitate the development of a novel innate T cell-based therapy for NEC.

## Author contributions

JL: Writing – original draft, Writing – review & editing. KM: Writing – review & editing. SJ: Writing – review & editing. JL: Writing – review & editing. JB: Writing – review & editing. CS: Writing – review & editing. TM: Writing – review & editing.
